# Risks Related to Chikungunya Infections among European Union Travelers, 2012–2018

**DOI:** 10.3201/eid2606.190490

**Published:** 2020-06

**Authors:** Céline M. Gossner, Nelly Fournet, Joana Gomes Dias, Beatriz Fernández Martínez, Martina Del Manso, Johanna J. Young, Hervé Zeller, Denis Coulombier

**Affiliations:** European Centre for Disease Prevention and Control, Solna, Sweden (C.M. Gossner, J. Gomes Dias, J.J. Young, H. Zeller, D. Coulombier);; Santé Publique France, Saint-Maurice, France (N. Fournet);; Instituto de Salud Carlos III, Madrid, Spain (B. Fernández Martínez);; Istituto Superiore di Sanita, Rome, Italy (M. Del Manso)

**Keywords:** chikungunya, chikungunya virus, viruses, risks, travel, travelers, surveillance, data review, outbreak, vector-borne infections, mosquitoes, zoonoses, European Union

## Abstract

Autochthonous outbreaks of chikungunya have occurred in the European Union (EU) after virus introduction by infected travelers. We reviewed the surveillance data of travel-related cases reported in the EU during 2012–2018 to document factors associated with increased infection rates among travelers and to assess how surveillance data could support preparedness against secondary transmission and timely control of outbreaks. Thirteen EU countries reported 2,616 travel-related chikungunya cases. We observed 3 successive epidemiologic periods; the highest number of cases (75%) occurred during 2014–2015, when most cases were associated with the Caribbean and South America. The highest infection rates among travelers were observed during the same phase. Although surveillance of travel-related cases is relevant for estimating the infection risk for travelers, we could not identify a relationship between the number of infected travelers and a higher likelihood of secondary transmission in the EU.

Chikungunya is a disease carried by *Aedes* mosquitoes that affects >100 countries, mostly in the tropics and subtropics (*1*). In the European Union (EU), chikungunya is not endemic, even though some outbreaks were reported in France and Italy after introduction of chikungunya virus by travelers into receptive areas (areas in which *Aedes albopictus* mosquitoes were established and active) (*2**–**6*).

To limit secondary transmission, it is crucial to monitor the distribution and activity of the mosquito vectors of the virus, reduce the likelihood of introduction of the virus by travelers, detect infections among returning travelers early, and implement timely control measures for cases in receptive areas. Consequently, surveillance of chikungunya was implemented during 2008 by the EU, whose goal was supporting these objectives (*7*).

We reviewed the surveillance data of travel-associated chikungunya cases reported in the EU during 2012–2018 with 2 aims. The first aim was to document factors associated with increased infection rates among travelers so that travelers, travel clinics, and public health authorities have relevant information to mitigate risks for infection. The second aim was to review how surveillance data could support preparedness against secondary transmission and timely control of outbreaks in susceptible areas.

## Methods

### Travelers

We obtained traveler data for 2012–2017 from the International Air Transport Association, which records passengers on commercial flights. We did not have access to 2018 data and considered it equal to 2017 data. We analyzed the number of travelers flying from chikungunya-affected countries to EU countries per month. We considered the departure and arrival countries irrespectively of connecting flights and assumed that case-patients were flying from the country in which infection occurred.

### Travel-Related Cases

We defined a travel-related case-patient as a person reported by an EU country, later called reporting countries, with a probable or confirmed chikungunya infection acquired outside their country of residence during 2012–2018. Cases were reported to the European Centre for Disease Prevention and Control (ECDC) (*8*). For time-related analysis we used, in order of preference, the date of onset, the date of diagnostics, or the date of notification. When none of these dates were available and if the date used for statistics was earlier than any of the dates mentioned, we used the date used for statistics, which is an unspecified, mandatory date.

We defined a probable case-patient as a person who had fever, returned from an area with ongoing chikungunya transmission within 2 weeks before onset of symptoms, and had virus-specific IgM in 1 serum sample (*9*). We defined a confirmed case-patient as a person satisfying any of the following laboratory criteria: detection of virus nucleic acid or virus isolation from a clinical specimen, virus-specific IgM in 1 serum sample plus confirmation by neutralization, or seroconversion or 4-fold antibody titer increase of specific antibodies in paired serum samples (*9*).

### Vector Distribution and Population

For each year, we obtained data on establishment of *Ae. albopictus* mosquitoes at the regional level (third level of the Nomenclature of Territorial Units for Statistics [*10*]) from the VectorNet database (*11*) and the French Ministry of Health website (*12*) and the population in reporting countries from Eurostat (*13*). We calculated the percentage of the population in regions in which the vector was established (population living in regions in which *Ae. albopictus* mosquitoes were established × 100/total population in the country). We grouped countries per geographic regions according to the United Nations Statistics Division definitions (*14*).

### Inclusion Criteria

The applied inclusion criteria (Appendix Figure) aimed to account for possible errors in gathering or reporting travel history/exposure of case-patients and lack of specificity of IgM serologic testing (*15**,**16*). We included probable and confirmed travel-related cases. Cases related to the French overseas territories (e.g., Martinique, French Polynesia) were considered as travel-related cases and those overseas territories as countries of infection. We also included reporting countries that submitted data every year and provided country of infection for >50% of their cases (arbitrary cutoff value) over the study period. When multiple countries of infection were reported for 1 case, the country of infection was changed to unknown. Finally, we included cases with a known country of infection; countries of infections that were associated with >2 cases, of which >1 was a confirmed case, and that were either reported by 2 reporting countries or reported over multiple years; and countries of infection that had travelers data available.

### Analysis

To obtain information that could support prevention of cases among travelers, we performed a descriptive analysis of travel patterns, case characteristics, reporting countries, and countries of infection. As a proxy of the risk for infection, we calculated infection rates among travelers (TIR = no. cases/100,000 travelers).

To define the risk for secondary transmission, we conducted a (nonsystematic) literature search on occurrences of secondary transmission in the EU during 2012–2018 and estimated the number of cases that could have led to autochthonous outbreaks. We considered that travel-related cases were distributed evenly within the reporting countries; for each year and reporting country, we multiplied the number of travel-related cases that occurred during June–October (when the vector is more abundant and active) by the percentage of population in regions where *Ae. albopictus* mosquitoes were established. We did not consider secondary transmission by donations of substances of human origin. We performed statistical analyses by using STATA/SE 14.0 software (https://www.stata.com) and Microsoft Excel 2016 (https://www.microsoft.com).

## Results

**Figure 1:**
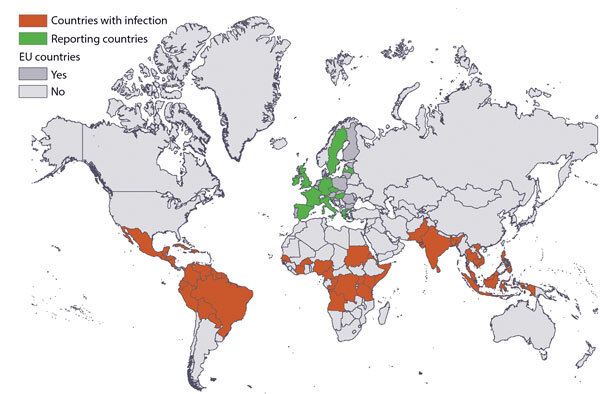
Risks related to chikungunya infections among EU travelers, 2012–2018. Countries with infection and reporting countries are indicated. Map produced on January 8, 2020. Administrative boundaries were obtained from EuroGeographics and the United Nations Food and Agriculture Organization. EU, European Union.

We identified 13 reporting countries and 59 countries of infection (Figure 1). During 2012–2018, a total of 146 million travelers arrived in reporting countries from countries of infection (Appendix Table). Most of these travelers arrived from Southeast Asia (27%), southern Asia (19%), and the Caribbean (15%). More specifically, 12% arrived from India, 12% from Thailand, and 8% from Brazil. The United Kingdom (31%), France (22%), and Germany (16%) received the highest number of travelers.

Travelers from the Caribbean (41%) and Polynesia (74%) arrived primarily in France. Travelers from South (32%) and Central (29%) America arrived mainly in Spain. Travelers from Southeast (32%) and southern (55%) Asia and from eastern Africa (44%) arrived primarily in the United Kingdom.

The overall yearly number of travelers increased during 2012–2018 from 18 million to 24 million. The highest increases in travelers during that period were for Northern Africa (88%), Central America (71%), and the Caribbean (54%). There were also large annual variations in number of travelers returning from specific countries of infection. For instance, the number of travelers from Venezuela decreased by 58% during 2013–2015 largely because of domestic insecurity and political crisis.

The number of EU travelers exhibited a seasonal pattern with peaks related to holiday periods (January, March, and August). Peak periods varied among reporting countries: March and August for France; January and March for Germany; July–September for Spain; January for Sweden; and January, April, and August for the United Kingdom.

### Risk for Infection among Travelers

During 2012–2018, there were 2,616 travel-related chikungunya cases, among which 1,766 were confirmed cases and 850 were probable cases (Table 1). France (918), the United Kingdom (593), Spain (587), and Germany (359) reported 94% of the cases. Although most reporting countries reported mostly confirmed cases, 60% of the cases reported by France and 31% of the cases reported by the United Kingdom were confirmed. The global TIR was 1.8 cases/100,000 travelers (Appendix Table).

**Table 1:** Characteristics of 2,616 persons with travel-related chikungunya infections, 2012–2018

Characteristic	No. case-patients (%)
Case classification	
Probable	850 (32)
Confirmed	1,766 (68)
Sex	
F	1,517 (58)
M	1,088 (42)
Unknown	11 (<1)
Age group, y	
<1–4	25 (1)
5–14	79 (4)
15–24	154 (7)
25–44	936 (43)
45–64	775 (35)
>65	221 (10)
Unknown	426 (16)
Year of infection	
2012	37 (1)
2013	45 (2)
2014	1,431 (55)
2015	448 (17)
2016	365 (14)
2017	171 (7)
2018	119 (6)
Month of infection	
January	133 (5)
February	89 (3)
March	96 (3)
April	150(5)
May	296 (11)
June	392 (15)
July	340 (13)
August	284 (11)
September	240 (9)
October	220 (8)
November	209 (8)
December	167 (6)

**Figure 2:**
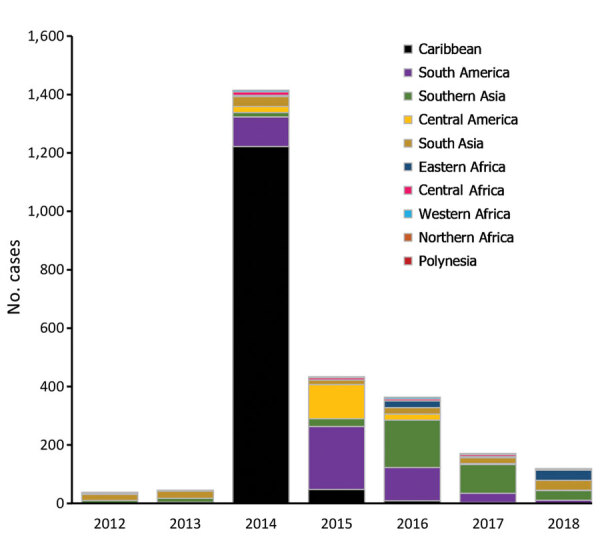
Number of travel-related chikungunya cases during 2012–2018, by region of infection and year.

The number of cases and global TIR fluctuated over the study period (Table 1; Figure 2; Appendix Table); both values peaked in 2014. There was a seasonal increase in cases during May–September, with a peak in May–June for Spain, May–August for France, September–November for Germany, and October–November for the United Kingdom. The global TIR was highest in May, June, and July. The median age of travel-related case-patients was 43 years (interquartile range 32–55 years), and the female-to-male ratio was 1.4:1.

**Figure 3:**
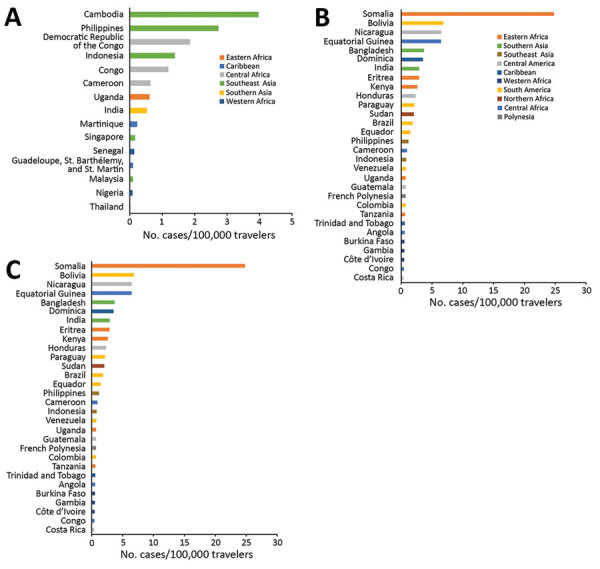
Rates of chikungunya infections among European travelers by country, region of infection, and epidemiologic period, 2012–2018. Shown are the 30 countries with the highest rates of infection for each period. A) 2012–2013; B) 2014–2015; C) 2016–2018.

**Figure 4:**
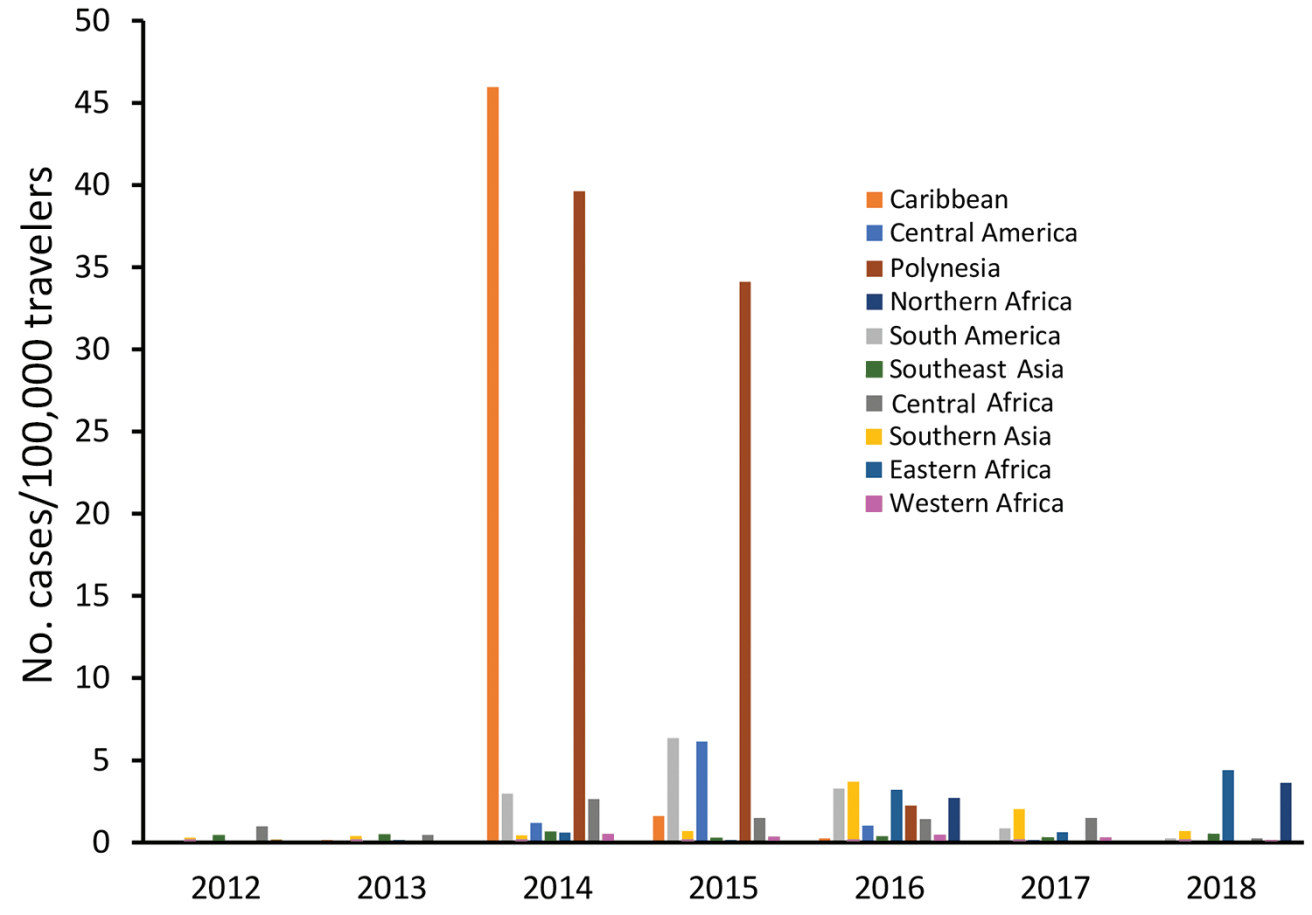
Rates of chikungunya infections among European Union travelers by region of infection and year, 2012–2018.

**Figure 5:**
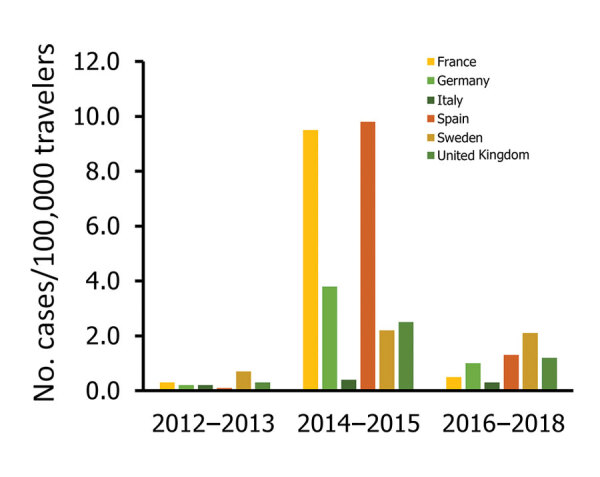
Rates of chikungunya infections among European Union travelers returning from countries of infection, by reporting country and epidemiologic periods, 2012–2018. Only countries reporting the highest number of cases were included.

We observed 3 successive epidemiologic periods (Figures 2,3,4,5). The first phase, 2012–2013, had few cases, most (84%) associated with southern and Southeast Asia; the global TIR was 0.2. In November 2013, one case was associated with the Caribbean, marking the start of epidemics in the Americas (*17*).

The second phase, 2014–2015, corresponded to the globalization of virus circulation; the global TIR was 4.8. Most cases were associated with the Caribbean (68%) and South America (17%). In 2014, the TIR for travelers from the Caribbean reached 45.9; in 2015 it decreased in travelers from the Caribbean (1.6), but it increased for travelers from South America (6.3) and Central America (6.1). In 2014, there was a high TIR for travelers returning from Haiti (266.6) and Dominica (193.6), and during 2015, a high TIR was associated with Honduras (78.1) and Nicaragua (76.5).

During the 2014–2015 phase, a high TIR was associated with Polynesia (36.8), which matched the intense epidemic in the region; in French Polynesia for instance, a few months after introduction of the virus, 25% of the population had been affected (*18*). In Africa, high TIRs were observed in travelers returning from Angola (12.0) during 2014 and Equatorial Guinea (9.2) during 2015. During that phase, travelers arriving in France and Spain had the highest TIRs: 9.5 for France and 9.8 for Spain.

The third phase, 2016–2018, was marked by a decrease in the global TIR. During that phase, a large percentage of case-patients were returning from southern Asia (45%) and South America (24%); regional TIRs were 2.1 for southern Asia and 1.4 for South America. During 2016, there were high TIRs for travelers returning from Bolivia (21.4) and Nicaragua (18.2). In Africa, high TIRs were seen for travelers from Somalia during 2016 (68.3) and Kenya during 2018 (6.6); a large epidemic affected Mombasa County in Kenya during 2018 (*19*). During 2016–2018, travelers arriving in Germany, Spain, Sweden, and the United Kingdom had TIRs ranging from 1.0 to 2.1, and travelers arriving in France had a TIR of 0.5.

### Risk for Secondary Transmission within the EU

During 2012–2018, a total of 3 autochthonous chikungunya outbreaks occurred in the EU. Two occurred in France in Hérault Department (September–October 2014) and Var Department (August 2017) (*3**,**4*). For the outbreak in Hérault, the index case-patient returned from Cameroon. For the outbreak in Var, the virus originated in Central Africa. One large outbreak occurred in the Lazio and Calabria regions of Italy (June–November 2017) (*5*). The index case-patient was not identified, but the virus originated in Pakistan or India (*6**,**20*).

During the study period, 10 case-patients returned from Cameroon (1–2/year). France reported the only case-patient during 2014; the TIR for travelers from France that year was 0.9. The highest TIR related to Cameroon was during 2017 for travelers from Spain (TIR = 28.5). During 2012–2018, a total of 38 case-patients were reported from Central Africa, 6 of them during 2017: 2 by France (TIR = 1.0) and 4 by Spain (TIR = 15.3). The highest TIRs related to Central Africa were in 2014 and 2017 among travelers from Spain (TIR = 17.3 during 2014 and 15.3 during 2017). During the study period, we detected 328 case-patients infected in India or Pakistan, 72 of them during 2017. Italy did not report any cases from these countries during 2017. During 2017, the TIR for travelers returning either from India or Pakistan was highest in the United Kingdom. The highest TIRs related to India or Pakistan were during 2016 among travelers from Malta and Slovenia. During 2014, travelers from France who had the highest TIRs (>600) were returning from Dominica, Suriname, and Tonga. During 2017, among travelers from France and Italy, those who had the highest TIRs returned from Bangladesh (TIR = 12.5 for travelers from France and 8.7 for travelers from Italy).

Among the reporting countries, France, Germany, Greece, Italy, Malta, Slovenia, and Spain had receptive areas (Table 2). The percentage of the population in regions in those countries colonized by *Ae. albopictus* mosquitoes increased during 2012–2018, from 28% in 2012 to 45% in 2018. We estimated that 270 travel-related case-patients returned to receptive areas during 20122018; 171 (63%) were in 2014. Among the estimated case-patients, 163 arrived in France, 79 in Spain, and 25 in Italy.

**Table 2:** Estimated number of travel-related cases of chikungunya that could have led to secondary transmissions on the basis of cases reported during June–October and proportion of population in countries in which *Aedes albopictus* mosquitoes are established, per year and reporting country, 2012–2018

Characteristic, year	France	Germany	Greece	Italy	Malta	Slovenia	Spain	Total
No. travel-related case during June–October								
2012	7	3	0	2	0	0	0	12
2013	6	0	0	3	0	0	1	10
2014	540	85	1	0	0	0	180	806
2015	35	29	0	9	0	0	87	160
2016	16	28	1	9	0	1	31	86
2017	10	11	0	3	0	0	14	38
2018	5	7	1	2	0	0	2	17
% Population in regions in which *Ae. albopictus* mosquitoes are established								
2012	21	0	6	83	92	11	20	28
2013	23	0	17	84	93	11	20	29
2014	25	0	25	86	93	14	20	31
2015	34	0	27	81	93	31	30	34
2016	36	0	76	99	93	57	33	41
2017	43	0	49	100	93	57	42	43
2018	55	1	83	100	93	57	42	45
Estimated no. cases in regions in which *Ae. albopictus* are established								
2012	1	0	0	2	0	0	0	3
2013	1	0	0	3	0	0	0	4
2014	135*	0	0	0	0	0	36	171
2015	12	0	0	7	0	0	26	45
2016	6	0	1	9	0	1	10	26
2017	4*	0	0	3*	0	0	6	13
2018	3	0	1	2	0	0	1	6
Total no. cases in regions in which *Ae. albopictus* are established								
2012–2018	163	0	2	25	0	1	79	270

*When an outbreak occurred.

## Discussion

We documented factors associated with increased travel-associated chikungunya cases reported in the EU during 2012–2018. Travel patterns (i.e., volume of travelers, country visited, and period of travel) were specific to each of the reporting countries. These patterns also reflect the geopolitical context, historical and cultural links, and preferences of travelers.

The TIR fluctuated according to the region/country visited, the reporting country, and the period of travel. The difference in TIR and seasonality among reporting countries was likely caused by difference in places visited within the countries of infection, intensity of virus circulation at the time of the visit, and reason for travel (e.g., business versus holidays). Generally, we observed a much higher TIR for travelers visiting a country with historical links to the reporting country (i.e., travelers from Spain visiting countries in Central America or travelers from France visiting French Overseas Countries and Territories). This finding might be explained by a high number of travelers visiting friends or relatives (VFR travelers). These travelers are less likely to receive pretravel advice than other types of travelers (*21*), might stay longer in the visited country, and might use fewer protective measures. Therefore, EU countries should consider issuing their own specific travel advice on the basis of countries most visited and those that have a higher risk for infection for their citizens. Prevention campaigns could be strengthened before and during peaks of cases and TIR, and be tailored to at-risk populations (e.g., VFR travelers). To reach these populations, travel advice could be provided online when flight tickets are purchased or through social media.

A high number of cases or high TIR might also highlight a sensitive surveillance system. This suggestion explains a slightly higher TIR among travelers from Sweden compared with other travelers during the 2012–2013 and 2016–2018 phases. With an overall number of travelers that is relatively low and a sensitive surveillance system, Sweden was able to test most possible case-patients and therefore detect a high proportion of cases among travelers.

As highlighted in other reports (*22*,*23*), travel-related cases are good indicators of the epidemiologic situation of the country visited because outbreaks are reflected by an increase in travel-related cases. Our results accurately highlighted the spread of the virus throughout the Americas, Polynesia, and eastern Africa. Therefore, travelers can be considered as sentinels, particularly in countries in which disease surveillance is limited (e.g., Somalia).

If there are few travelers, the likelihood of observing cases is limited. Therefore, having no cases associated with a specific country does not necessarily mean that no virus is circulating. It is useful to consider traveler data, and TIR provides a more accurate estimation of the risk for infection for travelers.

The higher proportion of female case-patients could be explained by the fact that women are at higher risk for development of severe symptoms and are therefore are more likely to seek medical attention (*24*). In addition, more female travelers might be exposed. For instance, in Spain, 61% of the travel-related cases were VFR travelers (*25*); those travelers are expected to be persons who emigrated to Spain from disease-endemic countries, mostly from the Americas. A high proportion of persons born in Central America/Caribbean (64.5%) and South America (55.6%) and living in Spain are women (*26*). Therefore, we might consider that more female travelers were going to the Americas as VFR travelers and became infected during that visit.

The number of cases and TIR do not seem to correlate with the likelihood of occurrence of autochthonous outbreaks in reporting countries and the origin of the imported outbreak strain. We found no autochthonous outbreaks associated with epidemics in the Americas during 2014–2015. In contrast, we found 2 autochthonous outbreaks during 2017 despite the relatively lower number of imported cases than in previous years. The autochthonous outbreaks that occurred in the EU were not consistently associated with regions/countries of infections with most cases or high TIRs. In addition, although we estimated that pressure of introduction in receptive areas in Spain was higher than that for those in Italy, no outbreaks were reported in Spain. Also, outbreaks have not occurred in years that had higher pressure of introduction. Many other factors account for allowing an autochthonous outbreak, including activity and abundance of the vector, environmental factors, adaptation of the virus strain to the local vector, surveillance sensitivity, and timeliness of control measures implemented for imported cases (*27**–**29*). Consequently, although monitoring of outbreaks worldwide is relevant for estimating the risk for infection to travelers, this monitoring does not seem to be as relevant for estimation of the risk for secondary transmission.

In the absence of valid data on vector competence of local *Ae. Albopictus* mosquito populations and the particular chikungunya virus strain, each imported case should be considered as a potential index case. Control measures should then be implemented for the case to limit the likelihood of virus and disease spread.

In the context of the global climatic and ecologic changes, it is expected that *Ae. albopictus* mosquitoes will colonize new areas of the EU, thus creating new areas at risk for local transmission (*30**,**31*). Furthermore, the *Ae. aegypti* mosquito, another competent vector, is threatening to establish itself on the continent, increasing even more the risk for local transmission (*32*).

Surveillance systems in EU countries are diverse and have evolved over time (e.g., chikungunya became a mandatory reportable disease in Spain during 2014). Detailed information about national surveillance systems is available in the ECDC annual epidemiologic reports (*33*). Large outbreaks worldwide increased awareness among physicians, potentially enhancing testing of potential cases. Consequently, comparisons between years and between reporting countries should be made cautiously.

Vector presence and activity in countries of infection vary at the local level, whereas we analyzed country level data. We did not have information about the reason for travel (e.g., tourism, business, VFR) and length of stay, 2 variables that are likely to influence the risk for infection (*34*,*35*). This limitation prevented us from identifying recommendations at the subnational level and targeted to the travelers categories.

The reported travel-related cases only represent a fraction of the actual infections because <25% of infected persons remain asymptomatic (*36*). In parallel, 34% of cases were probable cases, diagnosed with 1 IgM test, a highly unspecific test that leads to a high number of false-positive results and, in some instances, indicates a previous infection (*15**,**16**,**37**,**38*). These biases were likely to be constant over time and place and therefore did not affect interpretation of our results.

We considered that all reported case-patients had viremia while in the EU and therefore could have contributed to secondary transmission. However, a study published in 2007 estimated that 63% of infected travelers were viremic upon return to their home country (*39*), which would suggest that we overestimated the number of potential index cases.

Several studies that assessed risk for travelers related to chikungunya and dengue used travelers data from the United Nation World Tourism Organization (*22*,*40*) or national databases *41*,*42*). As highlighted in another report (*43*), there are major differences in the number of travelers between databases. The International Air Transport Association collects the number of flight passengers from 1 departure airport to an arrival airport, whereas other databases also include multicountry journeys and other types of travel mode, such as cruise ships; in addition, some databases might count travelers on the basis of their nationality instead of place of departure. We compared our results with those of 1 study that assessed the risk for chikungunya infection among travelers from Spain during 2008–2014 (*22*). Although results from the 2 studies are not in disagreement, they are not directly comparable. We believe that main difference in methods was that we used travelers data adjusted per year (except for 2018) and that the authors of the other study used mean annual number of travelers from previous years. If one considers major variations in number of travelers per year (e.g., Venezuela), we recommend researchers to used yearly number of travelers to estimate TIR. This recommendation emphasizes that comparison of TIRs between studies should be made with caution.

In conclusion, monitoring the number of travel-related chikungunya cases and the TIR among travelers can support travel advice. However, our results showed no indication that these factors can be useful in estimating the risk for autochthonous outbreaks in EU countries.

Appendix Additional information on risks related to chikungunya infections among European Union travelers, 2012–2018.
