# Supplementary material for: Risks Related to Chikungunya Infections among European Union Travelers, 2012–2018
Source: Emerg Infect Dis. 2020 Jun;26(6):1067–76. doi: 10.3201/eid2606.190490 (PMC7258487; doi:10.3201/eid2606.190490)
Supplement: Appendix — Additional information on risks related to chikungunya infections among European Union travelers, 2012–2018. [file 19-0490-Techapp-s1.pdf]

Article DOI: https://doi.org/10.3201/eid2606.190490

Risks Related to Chikungunya Infections among European Union Travelers, 2012–2018

Appendix

Appendix Table. Travelers returning from countries of infection, chikungunya cases and rates of infection among travelers, per reporting country and per region of infection, 2012–2018

Region of infection	Variable	Czech Republic	France	Germany	Greece	Hungary	Ireland	Italy	Latvia	Malta	Slovenia	Spain	Sweden	United Kingdom	Total
South America	No. cases	2	60	88	1	0	0	20	1	0	0	256	13	30	471
	% cases	0	13	19	0	0	0	4	0	0	0	54	3	6	100
	No. travelers	162,021	4,903,438	3,047,638	173,748	111,014	276,454	4,295,986	12,123	28,604	9,766	7,893,846	399,818	3,180,034	24,494,490
	% travelers	1	20	12	1	0	1	18	0	0	0	32	2	13	100
	No. cases/100,000 travelers	1.2	1.2	2.9	0.6	0.0	0.0	0.5	8.2	0.0	0.0	3.2	3.3	0.9	1.9
Central America	No. cases	1	17	42	0	0	0	6	1	0	0	75	8	14	164
	% cases	1	10	26	0	0	0	4	1	0%	0	46	5	9	100
	No. travelers	112,110	2,511,193	1,968,031	65,921	55,717	218,566	1,684,064	5,414	4,100	3,107	3,901,031	208,446	2,921,617	13,659,317
	% travelers	1	18	14	0	0	2	12	0	0	0	29	2	21	100
	No. cases/100,000 travelers	0.9	0.7	2.1	0.0	0.0	0.0	0.4	18.5	0.0	0.0	1.9	3.8	0.5	1.2
Caribbean	No. cases	3	723	113	1	0	0	1	0	0	0	208	10	229	1,288
	% cases	0	56	9	0	0	0	0	0	0	0	16	1	18	100
	No. travelers	72,741	9,174,056	3,275,191	124,203	51,204	184,373	1,842,732	7,197	5,918	2,999	2,565,094	166,131	5,093,514	22,565,353
	% travelers	0	41	15	1	0	1	8	0	0	0	11	1	23	100
	No. cases/100,000 travelers	4.1	7.9	3.5	0.8	0.0	0.0	0.1	0.0	0.0	0.0	8.1	6.0	4.5	5.7
Polynesia	No. cases	0	25	2	0	1	0	1	0	0	0	0	1	1	31
	% cases	0	81	6	0	3	0	3	0	0	0	0	3	3	100
	No. travelers	2,173	229,507	20,939	311	640	648	29,670	48	4	18	4,287	1,305	19,942	309,492
	% travelers	1	74	7	0	0	0%	10	0	0	0	1	0	6	100
	No. cases/100,000 travelers	0.0	10.9	9.6	0.0	156.3	0.0	3.4	0.0	0.0	0.0	0.0	76.6	5.0	10.0
Southeast Asia	No. cases	6	36	44	0	2	0	5	0	0	1	13	22	41	170
	% cases	4	21	26	0	1	0	3	0	0	1	8	13	24	100
	No. travelers	900,842	7,452,958	9,180,076	471,147	394,600	737,448	3,906,500	60,746	57,290	25,785	1,926,236	2,247,704	12,763,650	40,124,982
	% travelers	2	19	23	1	1	2	10	0	0	0	5	6	32	100
	No. cases/100,000 travelers	0.7	0.5	0.5	0.0	0.5	0.0	0.1	0.0	0.0	3.9	0.7	1.0	0.3	0.4
Southern Asia	No. cases	2	29	49	2	2	0	14	0	1	1	14	13	231	358
	% cases	1	8	14	1	1	0	4	0	0	0	4	4	65	100
	No. travelers	312,422	2,692,308	3,665,391	309,442	159,751	420,886	3,080,787	32,955	25,205	13,289	1,320,540	658,255	15,589,994	28,281,225

Region of infection	Variable	Czech Republic	France	Germany	Greece	Hungary	Ireland	Italy	Latvia	Malta	Slovenia	Spain	Sweden	United Kingdom	Total
	% travelers	1	10	13	1	1	1	11	%	0	0	5	2	55	100
	No. cases/100,000 travelers	0.6	1.1	1.3	0.6	1.3	0.0	0.5	0.0	4.0	7.5	1.1	2.0	1.5	1.3
Eastern Africa	No. cases	2	6	13	0	3	1	3	0	0	0	1	6	34	69
	% cases	3	9	19	0	4	1	4	0	0	0	1	%	49	100
	No. travelers	37,692	504,745	883,014	30,500	18,575	89,010	763,530	2,333	3,518	2,244	141,782	318,628	2,160,906	4,956,477
	% travelers	1	10	18	1	0	2	15	0	0	0	3	6	44	100
	No. cases/100,000 travelers	5.3	1.2	1.5	0.0	16.2	1.1	0.4	0.0	0.0	0.0	0.7	1.9	1.6	1.4
Central Africa	No. cases	0	16	6	0	0	0	0	0	0	0	14	0	2	38
	% cases	0	42	16	0	0	0	0	0	0	0	37	0	5	100
	No. travelers	5,900	1,618,405	240,661	11,459	5,699	16,992	317,675	3,622	1,814	1,080	203,446	21,987	531,186	2,979,926
	% travelers	0	54	8	0	0	1	11	0	0	0	7	1	18	100
	No. cases/100,000 travelers	0.0	1.0	2.5	0.0	0.0	0.0	0.0	0.0	0.0	0.0	6.9	0.0	0.4	1.3
Northern Africa	No. cases	0	0	1	0	0	0	0	0	0	0	0	0	2	3
	% cases	0	0	33	0	0	0	0	0	0	0	0	0	67	100
	No. travelers	1,758	18,934	56,647	7,915	2,849	10,549	34,015	141	964	166	6,200	31,009	109,720	280,867
	% travelers	1	7	20	3	1	4	12	0	0	0	2	11	39	100
	No. cases/100,000 travelers	0.0	0.0	1.8	0.0	0.0	0.0	0.0	0.0	0.0	0.0	0.0	0.0	1.8	1.1
Western Africa	No. cases	0	6	1	0	0	0	1	0	0	0	6	1	9	24
	% cases	0	25	4	0	0	0	4	0	0	0	25	4	38	100
	No. travelers	13,371	2,973,007	486,875	37,915	18,670	169,197	1,446,465	2,925	3,649	1,299	527,591	91,501	2,865,817	8,638,282
	% travelers	0	34	6	0	0	2	17	0	0	%	6	1	33	100
	No. cases/100,000 travelers	0.0	0.2	0.2	0.0	0.0	0.0	0.1	0.0	0.0	0.0	1.1	1.1	0.3	0.3
Total	No. cases	16	918	359	4	8	1	51	2	1	2	587	74	593	2,616
	% cases	1	35	14	0	0	0	2	0	0	0	22	3	23	100
	No. travelers	1,621,030	32,078,551	22,824,463	1,232,561	818,719	2,124,123	17,401,424	127,504	131,066	59,753	18,490,053	4,144,784	45,236,380	146,290,411
	% travelers	1	22	16	1	1	1	12	0	0	0	13	3	31	100
	No. cases/100,000 travelers	1.0	2.9	1.6	0.3	1.0	0.0	0.3	1.6	0.8	3.3	3.2	1.8	1.3	1.8

Supplemental file – Appendix figure. Inclusion criteria

All cases of chikungunya reported through The European Surveillance System between 2012 and 2018
Number of reporting countries: 19 (AT, BE, CZ, DE, EL, ES, FI, FR, HU, IE, IT, LV, MT, NL, PT, RO, SE, SI and UK)
Number of cases reported: 3581
Number of countries of infection: 92*

↓

Probable and confirmed travel-related cases
Number of reporting countries: 19 (AT, BE, CZ, DE, EL, ES, FI, FR, HU, IE, IT, LV, MT, NL, PT, RO, SE, SI and UK)
Number of cases reported: 3268
Number of countries of infection: 91

↓

Reporting countries that submitted data every year and provided country of infection for at least 50% of their cases over the study period
Number of reporting countries: 13 (CZ, DE, EL, ES, FR, HU, IE, IT, LV, MT, SE, SI and UK)
Number of cases reported: 3086
Number of countries of infection: 89

↓

Cases with known country of infections
Number of reporting countries: 13 (CZ, DE, EL, ES, FR, HU, IE, IT, LV, MT, SE, SI and UK)
Number of cases reported: 2666
Number of countries of infection: 89

↓

Countries of infections that were associated to at least two cases, of which one or more is a confirmed case, and that were either reported by two different reporting countries or reported over multiple years
Number of reporting countries: 13 (CZ, DE, EL, ES, FR, HU, IE, IT, LV, MT, SE, SI and UK)
Number of cases reported: 2621
Number of countries of infection: 60

↓

Countries of infection with travelers data available
Number of reporting countries: 13 (CZ, DE, EL, ES, FR, HU, IE, IT, LV, MT, SE, SI and UK)
Number of cases reported: 2616
Number of countries of infection: 59**

Note: AT (Austria), BE (Belgium), CZ (Czech Republic), DE (Germany), EL (Greece), ES (Spain), FI (Finland), FR (France), HU (Hungary), IE (Ireland), IT (Italy), LV (Latvia), MT (Malta), NL (The Netherlands), PT (Portugal), RO (Romania), SE (Sweden), SI (Slovenia) and UK (The United Kingdom)

** Afghanistan, Angola, Antigua and Barbuda, Argentina, Australia, Bangladesh, Barbados, Benin, Bolivia, Brazil, British Virgin Islands, Burkina Faso, Cambodia, Cameroon, Central African Republic, Colombia, Congo, Cook Islands, Costa Rica, Cuba, Democratic Republic of the Congo, Djibouti, Dominica, Dominican Republic, East Timor, Ecuador, Egypt, El Salvador, Equatorial Guinea, Eritrea, Ethiopia, Fiji, France, French Guiana, French Polynesia, Gambia, Ghana, Grenada and South Grenadines, Guadeloupe Saint Barthélemy and Saint Martin, Guatemala, Guyana, Haiti, Honduras, India, Indonesia, Italy, Ivory Coast, Jamaica, Kenya, Malawi, Malaysia, Maldives, Mali, Marshall Islands, Martinique, Mauritius, Mexico, Montserrat, Morocco, Mozambique, Myanmar, Nicaragua, Nigeria, Pakistan, Panama, Papua New Guinea, Paraguay, Peru, Philippines, Puerto Rico, Saint Lucia, Saint Vincent and Grenadines, San Marino, Senegal, Sierra Leone, Singapore, Sint Maarten, Somalia, Sri Lanka, Sudan, Suriname, Tanzania, Thailand, Togo, Tonga, Trinidad and Tobago, Uganda, United States, Venezuela, Vietnam, Western Samoa and Zimbabwe*

*** Angola, Bangladesh, Barbados, Bolivia, Brazil, Burkina Faso, Cambodia, Cameroon, Colombia, Congo, Costa Rica, Cuba, Democratic Republic of the Congo, Dominica, Dominican Republic, Ecuador, El Salvador, Equatorial Guinea, Eritrea, French Guiana, French Polynesia, Gambia, Grenada and South Grenadines, Guadeloupe Saint Barthélemy and Saint Martin, Guatemala, Guyana, Haiti, Honduras, India, Indonesia, Ivory Coast, Jamaica, Kenya, Malaysia, Martinique, Mexico, Nicaragua, Nigeria, Pakistan, Paraguay, Peru, Philippines, Saint Lucia, Saint Vincent and Grenadines, Senegal, Singapore, Sint Maarten, Somalia, Sri Lanka, Sudan, Suriname, Tanzania, Thailand, Tonga, Trinidad and Tobago, Uganda, Venezuela, Vietnam, and Western Samoa*

Appendix Figure. Inclusion criteria for study of risks related to chikungunya infections among European Union travelers, 2012–2018.
